# Iron deficiency anemia in pregnancy: prevention, diagnosis and treatment: Number 5 – 2026

**DOI:** 10.61622/rbgo/2026FPS5

**Published:** 2026-05-20

**Authors:** Carlos Alberto Maganha, Antonio Alceu dos Santos, Carolina Carvalho Mocarzel, Rone Peterson Cerqueira Oliveira, Isabel Cristina Céspedes, Rosiane Mattar

**Affiliations:** Faculdade de Ciências Médicas de São José dos Campos São José dos Campos SP Brazil Faculdade de Ciências Médicas de São José dos Campos, São José dos Campos, SP, Brazil.; Universidade Federal de São Paulo Escola Paulista de Medicina São Paulo SP Brazil Escola Paulista de Medicina, Universidade Federal de São Paulo, São Paulo, SP, Brazil.; Universidade do Estado do Rio de Janeiro Rio de Janeiro RJ Brzsil Universidade do Estado do Rio de Janeiro, Rio de Janeiro, RJ, Brzsil.; Universidade Federal da Bahia e Escola Bahiana de Medicina e Saúde Pública Salvador BA Brazil Universidade Federal da Bahia e Escola Bahiana de Medicina e Saúde Pública, Salvador, BA, Brazil.; Universidade Federal de São Paulo Escola Paulista de Medicina São Paulo SP Brazil Escola Paulista de Medicina, Universidade Federal de São Paulo, São Paulo, SP, Brazil.; Universidade Federal de São Paulo Escola Paulista de Medicina São Paulo SP Brazil Escola Paulista de Medicina, Universidade Federal de São Paulo, São Paulo, SP, Brazil.

## Key points

- Anemia affects 30% of the world's population, with iron deficiency identified as the main cause, accounting for up to 60% of cases.
- The prevalence of iron deficiency in pregnant women reaches 35% globally and 46% in low-income countries.
- Iron deficiency anemia is associated with higher maternal morbidity, mortality, and adverse perinatal outcomes (prematurity, low fetal weight).
- Iron supplementation during pregnancy and the postpartum period is fundamental in preventing anemia.
- Treatment of iron deficiency anemia with iron supplementation reduces mortality, transfusions, and hospital costs, and should be predominantly carried out with oral iron replacement.
- Treatment of iron deficiency anemia with injectable iron should be reserved for specific situations, such as intolerance to oral therapy, pregnancies beyond 34 weeks with low hemoglobin (Hb), Hb ≤ 8 g/dL, scheduled surgeries, and inadequate response to oral therapy.
- Erythropoietin is safe during pregnancy and may be an alternative in conjunction with injectable iron in specific situations.
- Although blood transfusion is not a therapeutic option for iron deficiency anemia, in rare and severe cases of gestational anemia, the attending physician may assess the risk of transfusion support. Importantly, this modality does not replenish the body's iron reserves.

## Recommendations

- Hemoglobin levels are lower during pregnancy due to gestational hemodilution. Normal Hb is considered to be ≥ 11 g/dL in the first and third trimesters, and ≥ 10.5 g/dL in the second trimester.
- In low-risk pregnancies, routine testing, such as serum ferritin and/or transferrin saturation, is not recommended to assess iron stores.
- Iron supplementation should be prescribed for all pregnant women after diagnosis and up to three months postpartum. The prescribed dose is 30 to 60 mg of elemental iron, administered once daily on an empty stomach. In cases of intolerance, a weekly dose of 120 mg of elemental iron may be prescribed.
- The diagnosis of anemia in pregnancy is defined when Hb < 11.0 g/dL.
- Iron deficiency anemia is considered when low iron stores are associated with anemia, preferably diagnosed by serum ferritin less than 30 ng/mL.
- Another test that can be performed and defines low iron stores is transferrin saturation < 15%.
- Treatment of iron deficiency anemia is preferably carried out by oral replacement of elemental iron at a daily dose of 2 to 5 mg/kg (equivalent to 100 to 200 mg), administered into two or three divided doses, until normalization of Hb values (after one to two months) or serum ferritin exceeds 30 ng/mL (after two to six months).
- The oral iron compound chosen for supplementation and/or replacement can be the one that best suits the economic conditions and acceptability of the patient, respecting the amount of elemental iron in each compound.
- Parenteral treatment should preferably be intravenous (IV) and reserved for special cases, such as: intolerance to oral iron therapy; anemia requiring rapid normalization (advanced pregnancy > 34 weeks), regardless of Hb level; anemia with Hb ≤ 8.0 g/dL (evaluate the association with erythropoietin); scheduled surgeries within 30 days with a risk of bleeding > 500 mL in pregnant women with anemia; inadequate response to oral iron therapy (insufficient increase in Hb levels < 1.0 g/dL in 14 days), and especially in the presence of inflammatory bowel disease, gastric surgery, and chronic kidney disease.
- The combination of erythropoietin (EPO) with IV iron is recommended as treatment in cases of resistant anemia or anemia not resolved with iron supplementation alone. Its main indications are: anemia in patients with gestation > 34 weeks who do not respond to iron alone (oral or IV); anemia with Hb < 9.5 g/dL; anemia in patients with chronic renal failure (glomerular filtration rate < 60 mL/min/1.73 m^2^ or on dialysis).
- Although blood transfusion is not recommended for treating iron deficiency anemia, the attending physician may assess the need for transfusion support in severe, exceptional, or acute scenarios requiring immediate Hb replacement.
- The gestational age at delivery, as well as the route of delivery, is not affected by iron deficiency anemia and/or its clinical treatment.

## Background

The prevalence of anemia worldwide is around 30%. Iron deficiency is the predominant etiology, responsible for approximately 60% of cases^(1)^ and 22% of maternal deaths in 2019.^(2)^

Around 35% of pregnant women worldwide have iron deficiency. This rate increases to 46% when lower-income countries are evaluated, where malnutrition during pregnancy is more prevalent.^(3,4)^ Anemia is an independent risk factor for increased postoperative morbidity and mortality^(5)^ and the greater the severity of the patient's anemia, the greater the risk of mortality.^(6)^ Minimizing allogeneic blood transfusions is associated with better clinical outcomes (reduction in mortality, myocardial infarction, stroke and infection, and shorter hospital stay) and lower hospital costs.^(7)^ Treating anemia with iron replacement and/or erythropoiesis-stimulating agents results in reduced mortality.^(8)^ Given this evidence, it is necessary to detect and diagnose gestational anemia and malnutrition early. The objective of this document is to provide healthcare professionals with actionable scientific strategies for iron supplementation and the correction of anemia during pregnancy.

## Does pregnancy interfere with a woman's hematological system? How to interpret laboratory parameters?

In pregnancy, there is a physiological expansion of plasma volume beginning in the first trimester and stabilizing in the third, which exceeds the increase in red blood cell and Hb production. The resulting hemodilution contributes to the drop in Hb during pregnancy. Several factors can restrict or reduce this expansion, such as preeclampsia.^(4)^

Anemia is conventionally identified when the Hb concentration falls below a defined limit. The World Health Organization (WHO) defines anemia when the Hb concentration is less than 11 g/dL in the first and third trimesters and less than 10.5 g/dL in the second trimester. It also stratifies the severity of anemia according to table 1.^(9)^

**Table 1:** Hemoglobin cut-off points in pregnancy according to the World Health Organization

Pregnancy	Absence of anemia	Mild anemia	Moderate anemia	Severe anemia
1^st^ trimester	≥11 Hb	10-10.9 Hb	7.0-9.9 Hb	<7.0 Hb
2^nd^ trimester	≥10.5 Hb	9.5-10.4 Hb	7.0-9.4 Hb	<7.0 Hb
3^rd^ trimester	≥11 Hb	10-10.9 Hb	7.0-9.9 Hb	<7.0 Hb

Source: Adapted from World Health Organization (2024).^(9)^

Hb: hemoglobin;

* Hb in g/dL

However, from a practical point of view, Hb ≥ 11 g/dL is considered normal.^(10,11)^

## Is iron supplementation necessary in a normal pregnancy to prevent anemia?

Iron utilization increases during pregnancy, as it is necessary for fetal growth and development, as well as for increased maternal erythropoiesis.^(4)^ In pregnancy, iron deficiency is usually due to an imbalance between demand and supply, which worsens as the pregnancy progresses.^(4)^ Anemia can have long-term consequences, but specifically in pregnancy it is associated with an increased risk of cesarean section and maternal mortality, and may contribute to adverse outcomes in the newborn, including reduced birth weight and gestational age.^(9–11)^ Routine elemental iron supplementation is a controversial subject in the literature.^(12)^ However, the Ministry of Health's high-risk pregnancy manual recommends iron supplementation for virtually all pregnant women, except those with diseases characterized by iron overload, for example, hemolytic anemias and hemochromatosis.^(10)^ Iron supplementation should be prescribed for all pregnant women after diagnosis and up to three months postpartum. The recommended dose is 30 to 60 mg of elemental iron, administered once daily on an empty stomach.^(13)^ The WHO recommends varying doses throughout pregnancy. Initially, an oral dose of less than 30 to 60 mg of elemental iron is advised daily.^(14)^ From the second trimester of pregnancy (week 20) through three months postpartum, this dose should increase to 60 to 100 mg daily for all pregnant women, particularly for women with ferritin values below 30 ng/mL.^(4,10,11)^ The WHO also recommends, as an alternative, in selected cases of gastrointestinal intolerance or exclusion of iron deficiency (through Hb measurement), the use of 120 mg once a week.^(14)^ For adequate effectiveness of supplementation, it is ideal that the iron compound be ingested between meals or before bedtime. The absorption of oral iron is enhanced when co-administered with vitamin C or acidic foods, or when taken on an empty stomach (at least 60 minutes before meals). Conversely, antacids, H2 blockers, proton pump inhibitors, coffee and calcium (milk and dairy products) reduce oral iron absorption, as does the use of multivitamins and minerals, since divalent ionic metals (such as zinc, copper, and manganese) compete with each other for gastrointestinal absorption.^(10,11)^

## When to suspect that a pregnant woman has iron deficiency anemia and how to make the diagnosis?

**Figure 1:**
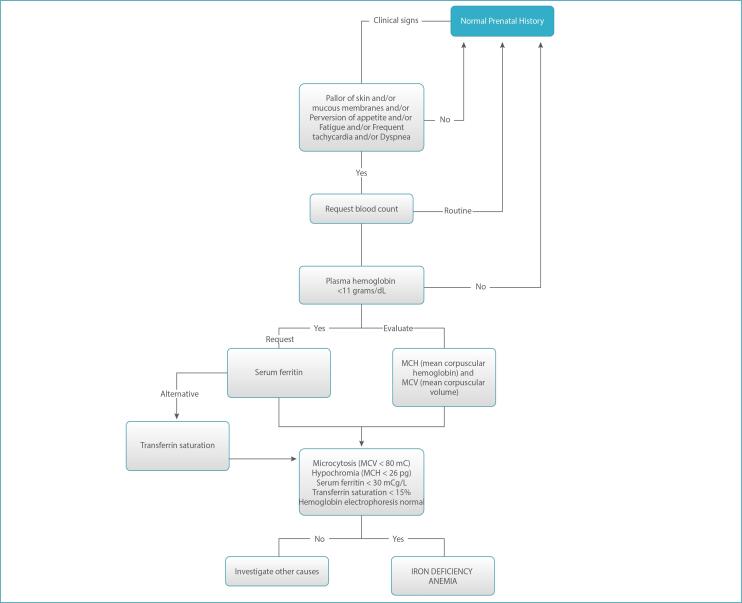
Flowchart for the diagnosis of iron deficiency anemia

Source: Maganha (2023).^(16)^

In cases of mild to moderate anemia, pregnant women are usually oligosymptomatic, but in severe cases there may be symptoms such as pallor of the skin and mucous membranes, fatigue, asthenia, loss of appetite, tachycardia and/or dyspnea without pulmonary or cardiac pathology, and a systolic murmur may be present at the mitral focus (physiological).^(10,11)^ In almost all clinical scenarios, the diagnosis of iron deficiency anemia will be based on blood count findings. The evaluation of hematimetric indices, especially mean corpuscular volume (MCV) and mean corpuscular hemoglobin (MCH), is very useful in the syndromic diagnosis of anemias. The definitive diagnosis of gestational anemia is made by detecting Hb < 11 g/dL, associated or not with symptoms.^(10,11)^ Once the diagnosis of gestational anemia is confirmed, it is necessary to detect serum iron stores to establish the clinical scenario of iron deficiency anemia. This anemia condition, as a rule, manifests with microcytosis (MCV < 80 μm) and hypochromia (MCH < 26 pg), thus being called hypochromic microcytic anemia. However, in the initial phases, microcytosis and hypochromia may not be present.^(10,11)^ Although serum ferritin is the most commonly used test to assess the pregnant woman's iron stores, its routine measurement is not recommended by the International Federation of Gynecology and Obstetrics (FIGO) for all pregnant women,^(15)^ but only when there is a picture of anemia. Low serum ferritin (<12 ng/mL) is a clear sign of iron deficiency in pregnant women. However, a normal ferritin level does not exclude iron deficiency, as pregnancy is associated with a physiological increase in acute phase proteins and changes in iron utilization and metabolism, both influencing serum ferritin levels.^(4)^ Therefore, the suggestion is to consider ferritin levels < 30 ng/mL, which are compatible with low iron stores and mandatory supplementation.^(10,11)^ Another test that can be used, but without evidence in pregnancy, is transferrin saturation. In iron deficiency anemia, it will be less than 15%.^(10,11)^ To organize and facilitate the diagnosis of iron deficiency anemia in pregnancy, we present the flowchart developed by Febrasgo's National Specialized Commission on High-Risk Pregnancy (Figure 1).^(16)^

## What are the main differential diagnoses of iron deficiency anemia in pregnancy?

The main cause of anemia in pregnancy is iron deficiency anemia.^(4,10–12)^ However, it is of utmost importance to rule out other causes, such as nutritional deficiencies (vitamin B12 and folate), hemoglobinopathies (sickle cell anemia, thalassemia and others), infections (malaria, schistosomiasis, hookworm disease), drug-induced anemia, and clinical conditions such as gynecological bleeding, gastrointestinal diseases, frequent blood donation, and chronic diseases that lead to blood loss or destruction of red blood cells.^(9)^ The observation of increased MCV (>100 μm) suggests the possibility of megaloblastic anemia, usually caused by vitamin B12 deficiency, but may be a consequence of folic acid deficiency. The measurement of these elements confirms the diagnosis.^(3,10,11)^ In cases of very low MCV (<70 μm), there is a possibility of anemia due to hemoglobinopathies, including thalassemia. Requesting Hb electrophoresis, with a possible increase in HbA2 and/or HbF, corroborates this diagnosis. Electrophoresis will also help rule out sickle cell anemia, in which HbS will be present in homozygosity or heterozygosity. The C-reactive protein measurement can help in recognizing anemias related to underlying infectious processes.

Glucose-6-phosphate dehydrogenase deficiency is a hereditary genetic disorder that causes red blood cells to break down prematurely, which can lead to anemia. This deficiency is one of the most common hereditary enzymatic abnormalities in humans.^(9)^ The proportion of anemia due to genetic disorders in low- and middle-income countries will likely increase as other causes (e.g., nutritional deficiencies, infectious diseases) become progressively better controlled.^(9)^ Hemoglobin concentrations are reduced by acute and chronic diseases. In some clinical scenarios of inflammation, autoimmune diseases, infection, cancer, heart failure, and even obesity, a reduction in erythropoiesis is observed due to functional iron deficiency and also reduced red blood cell survival.^(18)^ In renal failure, Hb concentrations are reduced due to functional iron deficiency, but fundamentally due to the reduced production of erythropoietinogen, a precursor of EPO, a glycoprotein that stimulates red blood cell production. Many medications (antibiotics, anti-inflammatories, and/or chemotherapeutic agents) can reduce Hb concentrations idiosyncratically or in a dose-dependent manner.^(18)^ Other clinical conditions, such as the presence of hypoxia (e.g., smoking, respiratory disease, obesity, sleep apnea, or high altitude of residence), can increase Hb concentrations.^(18)^

**Table 2:** Characteristics of serum laboratory findings according to the type of anemia

Diagnosis	Hemoglobin	Ferritin	Transferrin saturation	MCH	MCV
Functional iron deficiency	Normal	↓	↓	↓	Normal
Iron deficiency anemia	↓↓	↓↓	↓↓	↓↓	↓
Anemia due to vitamin B12 or folic acid deficiency	↓	Normal	Normal	↑	↑
Anemia due to infection	↓	Normal or ↑	↓	Normal	Normal
Hemoglobinopathies	↓↓	Normal or ↑	Normal or ↑	↓↓	↓↓

MCH: mean corpuscular hemoglobin; MCV: Mean corpuscular volume

Source: Adapted from Breymann (2000).^(17)^

## During pregnancy, what tests should be ordered, how often, and how should they be interpreted?

The most prevalent recommendation among societies and consensus guidelines stipulates performing a complete blood count, with evaluation of MCV and MCH at the beginning of pregnancy and the beginning of the third trimester (28 weeks), or when there is any complication between these periods.^(4)^ The interpretation of Hb and/or hematocrit (Ht) concentration has already been discussed (Table 1) and takes into account the hemodilution of pregnancy. Recently, the US Preventive Services Task Force (USPSTF) found no evidence to support the routine and systematic performance of complete blood counts in pregnant women who are not adolescents and do not show symptoms of anemia.^(19)^ However, there is considerable evidence in the literature that treating anemia improves clinical outcomes.^(8)^ In low-risk pregnancies, there is no evidence to support routine serum ferritin measurement.^(4,10,11)^ However, in the presence of anemia, it is the primary test to measure the mother's iron reserves and may be necessary for the differential diagnosis of other causes of anemia during pregnancy. Although transferrin saturation measurement is also not routinely requested, it can be an alternative for diagnosing iron deficiency anemia in strongly suspected cases with inconclusive ferritin measurements.

## How to treat iron deficiency anemia with oral iron supplementation in the different phases of pregnancy and the postpartum period?

Treatment of iron deficiency anemia should prioritize oral iron replacement whenever possible, as it is safer, more practical and less costly; however, it is often slower and less effective.^(20)^ In anemia scenarios (Hb < 11 g/dL), the usual dose of elemental iron orally should be 2-5 mg/kg daily, i.e., a daily dose ranging from 100 to 200 mg divided into two or three doses, until normalization of Hb values (after one to two months) and serum ferritin (>30 ng/mL — after two to six months). The dose and duration of iron replacement will vary depending on the depletion of iron stores, age, time, side effects, and the cause of anemia.^(21,22)^ Dividing the dose decreases the saturation of iron absorption per dose and improves the effectiveness of treatment.^(23)^ Recent studies suggest that alternate dosing of iron salts may offer superior efficacy and improved tolerability;^(24)^ however, further evidence is required before this approach can be standardized in clinical guidelines. One explanation is that administering high doses of iron induces the production of hepcidin, which, in turn, causes a decrease in iron absorption, a fact that would justify the use of iron on alternate days. Oral iron absorption is enhanced when co-administered with vitamin C and acidic foods and/or when taken on an empty stomach (at least 60 minutes before meals). Antacids, H2 blockers, proton pump inhibitors, coffee, and calcium (milk and dairy products) reduce oral iron absorption, as does the use of multivitamin-mineral supplements, since divalent ionic metals (such as zinc, copper, and manganese) compete with each other for gastrointestinal absorption. To facilitate prescription, we have prepared a table of iron compounds and their respective amounts of elemental iron (Table 3).

**Table 3:** List of available iron compounds, their elemental iron composition, and daily dose in the treatment of iron deficiency anemia

Iron salt	Presentation	Elemental iron	Daily dose (treatment)
Ferrous sulfate (20% elemental iron)	Coated tablets: 200 mg	40 mg	4-5 coated tablets
	Coated tablets: 300 mg	60 mg	3 coated tablets
	Coated tablets: 500 mg	100 mg	2 coated tablets
	Drops: 125 mg/mL	25 mg/mL	2 drops/kg weight
Iron(III) hydroxide polymaltose or ferripolimaltose (30% elemental iron)	Tablet: 435 mg	123 mg	2 tablets
	Chewable tablet: 330 mg	100 mg	2 tablets
	Oral solution: 330 mg/mL	100 mg/mL	1 mL/5 kg weight
	Drops: 182 mg/mL	50 mg/mL	1 drop/kg weight
Iron chelate glycinate or ferric glycinate (20% elemental iron)	Tablet: 150 mg	30 mg	5 tablets
	Tablet: 300 mg	60 mg	3 tablets
	Chewable tablet: 500 mg	100 mg	2 tablets
	Oral vials: 250 mg/5 mL	50 mg/5 mL	4 oral vials
	Drops: 250 mg/mL	50 mg/mL	1 drop/kg weight
Carbonyl iron (33% elemental iron)	Coated tablet: 400 mg	120 mg	2 coated tablets
Ferrous fumarate (33% elemental iron)	Tablet: 200 mg	60 mg	3 tablets
Gluconate Ferrous (12% elemental iron)	Tablet: 300 mg	36 mg	6 tablets

Source: Adapted from Arruda and Figueiredo (2013)^(25)^ and Cançado et al. (2010).^(26)^

Treatment efficacy is monitored by assessing Hb levels via a complete blood count; these should be monitored every 15-30 days, depending on the severity of anemia and gestational age. After initiating treatment with oral iron replacement, Hb is expected to increase by at least 0.3 g/dL each week. Once Hb enters the normal range, oral iron replacement needs to be maintained for another three months and at least six months postpartum. The most common adverse effects related to oral iron are gastrointestinal: constipation, diarrhea, epigastric pain, nausea, and vomiting.

## How and when to treat iron deficiency anemia with parenteral iron supplementation during pregnancy?

Parenteral iron replacement, preferably IV, is considered more effective and better tolerated than oral iron. Thus, IV administration results in faster correction of iron stores, better adherence to treatment, and improved quality of life.^(27,28)^ However, it should be reserved for specific situations, as it is more expensive and requires administration infrastructure. The main indications for IV therapy are: intolerance to oral iron therapy; anemia requiring rapid normalization (advanced pregnancy > 34 weeks), regardless of anemia severity; anemia with Hb ≤ 8 g/dL in patients with less than 28 weeks of pregnancy due to iron deficiency (ferritin < 30 ng/mL); scheduled surgeries within 30 days with a risk of bleeding > 500 mL in pregnant women with anemia; inadequate response to oral iron therapy (insufficient increase in Hb levels < 1 g/dL in 14 days), especially in the presence of inflammatory bowel disease, gastric surgery, and chronic kidney disease; supplemental therapy with EPO.^(4,29,30)^

**Chart 1:** Indications for intravenous iron replacement in pregnancy

Intolerance to oral therapy
Iron deficiency anemia > 34 weeks (requires rapid normalization)
Iron deficiency anemia with Hb ≤ 8 g/dL
Iron deficiency anemia with scheduled surgeries within 30 days with risk of bleeding > 500 mL (includes childbirth)
Inadequate response to oral therapy: Hb elevation < 1 g/dL in 14 days of treatment

Hb: hemoglobin

Intravenous iron replacement in our country can be performed using three compounds:^(19)^

- Iron hydroxide saccharate: should be administered at a dose of 200 mg/day, equivalent to two ampoules diluted in 200 mL of 0.9% saline solution, in slow infusion (one to two hours), with a maximum dose of 600 mg/week.
- Note: Total elemental iron replacement can be calculated using Ganzoni's formula: total iron = [Target Hb (g/dL) - Actual Hb (g/dL)] x body weight (kg) x 2.4 + 500.^(31)^
- Ferric carboxymaltose: dose of 20 mg/kg, approximately 1,000 mg (two ampoules) per week diluted in 200 mL of 0.9% saline solution, infused over 15 minutes in a single weekly dose.
- Ferric derisomaltose: dose of 20 mg/kg, which can be administered as 1,000 mg/week, diluted in 20 mL of 0.9% saline solution, infused over 15 minutes or as a 500 mg bolus infusion up to 3 times/week.

After treatment is successful (Hb ≥ 11 g/dL), prophylactic oral iron supplementation is recommended for up to six months postpartum.

The main contraindications to the use of IV iron are: patients with less than 16 weeks of gestation; presence of active infection/septicemia associated with bacteremia (in these cases, assess risk versus benefit and use with caution); anemia not related to iron deficiency (transferrin saturation greater than 45%, ferritin greater than 500 ng/mL); severe liver dysfunction; patients with potential secondary iron overload (hereditary hemolytic anemias, myelodysplastic syndrome, Fanconi anemia, and others) and pre-existing anaphylactic crisis.^(32,33)^

Intravenous iron administration is safe and does not appear to be associated with a significant increase in side effects, infections and/or mortality,^(34,35)^ but ideally the infusion should be performed in a hospital setting or, at least, in a health unit with a doctor present. Treatment with IV iron takes about seven days to raise Hb by 1 g/dL and about 3-4 weeks to 2 g/dL.^(36)^

Possible adverse effects with IV iron administration are: hypotension, urticaria, metallic taste sensation, skin rashes and anaphylaxis (rare event). In cases IV iron hypersensitivity, one established protocol includes: IV dexamethasone (10 mg); inhaled ipratropium bromide (0.5 mg) and salbutamol (2.5 mg); normal saline solution (60 mL/hour); and subcutaneous epinephrine (0.1 mg) at the start of the iron infusion - with 0.3 mg of epinephrine maintained at the bedside for rescue use.^(37)^

## Can the treatment of iron deficiency anemia be associated with the administration of erythropoietin to accelerate recovery from severe anemia during pregnancy?

Erythropoietin therapy is safe in pregnancy both for the mother and the fetus, regardless of gestational age,^(38)^ with no reports of thromboembolism.^(4)^ Erythropoietin combined with IV iron is a treatment alternative in cases of resistant anemia or anemia not resolved with iron supplementation alone.^(39)^ The main indications for the use of EPO in the treatment of gestational anemia are: anemia in patients with gestation > 34 weeks who do not respond to iron alone (oral or IV); anemia with Hb < 9.5 g/dL; anemia in patients with chronic renal failure (glomerular filtration rate < 60 mL/min/1.73 m2 or on dialysis).^(39–41)^

*Note: Anemias associated with hematological diseases and/or anemias that do not respond to treatment (absence of Hb elevation) after 2-3 weeks with IV iron + EPO should be monitored by a hematologist.*

The recommended doses of EPO in gestational anemia are:^(38,39,41,42)^

- EPO 300 UI/kg/week, subcutaneous or IV; reassess in one week; if there is an increase in Hb, maintain this dose until Hb ≥ 10 g/dL.
- EPO 600 UI/kg/week, subcutaneous or IV, if there is no increase in Hb and in the presence of anemia (Hb < 9.5 g/dL) and/or pregnancy > 34 weeks.

*IMPORTANT NOTE: EPO has very low efficacy in an iron deficiency setting, therefore, in iron deficiency anemia scenarios, iron supplementation should always precede treatment with EPO.*

## Currently, are there indications for blood transfusion in the treatment of iron deficiency anemia?

We have no evidence in the current literature to treat iron deficiency anemia with allogeneic blood transfusion. However, in rare clinical scenarios of severe gestational anemia, the physician responsible for the patient may assess the risk of transfusion support, considering that this therapy only contributes to raising Hb levels, but does not correct body iron deficiency, and IV treatment with associated iron and EPO should be rapidly instituted.^(43,44)^

## What are the recommendations for prenatal obstetric follow-up of a pregnant woman with iron deficiency anemia?

Once the diagnosis of iron deficiency anemia is confirmed, therapeutic measures to replace this element should be initiated immediately to avoid maternal and fetal repercussions. The presence of symptoms, the severity of anemia, iron deficiency, and the existence of fetal repercussions will influence the interval between consultations to be adopted and the frequency of post-treatment control laboratory tests and fetal assessment tests. Iron deficiency should not influence the route of delivery, which will follow traditional obstetric indications. It is important to try to maximize iron stores and correct anemia before delivery, since patients with these conditions are more prone to complications in case of postpartum hemorrhage*.*^(4,45)^ Knowledge of pre-delivery Hb can guide the adoption of care such as ensuring peripheral access, an appropriate place for delivery, blood product reserves, in addition to general hemorrhage prevention measures adopted universally.^(28)^ Knowledge of the Hb value 48 hours after delivery is suggested for women with blood loss > 500 mL, those with uncorrected anemia in the antenatal period, or for patients with symptoms suggestive of postpartum anemia, as a way to guide the appropriate therapy to be implemented.^(4)^

## Final considerations

Iron deficiency anemia is a highly prevalent condition during pregnancy, with a significant clinical impact on both maternal and fetal health. It is associated with an increased risk of complications such as cesarean delivery, prematurity, low birth weight, and maternal mortality. Universal iron supplementation is recommended for virtually all pregnant women, from the second trimester until the postpartum period. Early detection of iron deficiency and timely diagnosis of iron deficiency anemia are fundamental for preventing adverse outcomes. A complete blood count, with special attention to Hb levels, and serum ferritin levels are the main tests used for screening and diagnosing this condition. Treatment should prioritize the oral route, as it is safer and less expensive, although in specific situations — such as gastrointestinal intolerance, the need for rapid correction, or failure to respond to oral treatment — IV iron administration may be indicated. In addition, EPO can be used as an adjunct in cases of severe or refractory anemia, especially in the presence of comorbidities such as renal insufficiency. Blood transfusion, however, is not recommended in the treatment of iron deficiency anemia. In obstetric follow-up, the delivery method should follow traditional obstetric criteria and not be modified by the presence of anemia. However, monitoring of Hb levels before and after delivery is recommended, especially in cases with significant blood loss or previously uncorrected anemia.

## Data Availability

research data are available in the article.

## National Specialized Commission on High-Risk Pregnancy of the Brazilian Federation of Gynecology and Obstetrics Associations (Febrasgo)

President:

Rosiane Mattar

Vice-president:

Inessa Beraldo de Andrade Bonomi

Secretary:

Mylene Martins Lavado

Members:

Carlos Alberto Maganha
Carolina Carvalho Mocarzel
Elton Carlos Ferreira
Fernanda Santos Grossi
Janete Vettorazzi
Maria Rita de Figueiredo Lemos Bortolotto
Renato Teixeira Souza
Rone Peterson Cerqueira Oliveira
Sara Toassa Gomes Solha
Vera Therezinha Medeiros Borges
Willian Schneider da Cruz Krettli
